# Functional Analysis of the Yeast Counterpart to the Human GCN2 p.Glu738_Asp739insArgArg Variant

**DOI:** 10.17912/micropub.biology.001767

**Published:** 2025-08-12

**Authors:** Anja H Schiemann, Makhdoom Sarwar, Evelyn Sattlegger

**Affiliations:** 1 School of Food Technology and Natural Sciences, Massey University, Palmerston North, New Zealand; 2 Maurice Wilkins Centre for Molecular BioDiscovery, University of Auckland, Auckland, New Zealand; 3 Biomolecular Interaction Centre, University of Canterbury, Christchurch, New Zealand; 4 Department of Obstetrics and Gynaecology, University of Otago, Christchurch, 2 Riccarton Avenue, Christchurch 8011, New Zealand

## Abstract

GCN2, a cytoplasmic protein kinase, helps cells adapt to nutrient deprivation and is implicated in various cancers. The GCN2 p.Glu738_Asp739insArgArg variant was found to be significantly enriched in a cohort of early-onset colorectal cancer cases, indicating possible lineage-associated predisposition. Introducing an equivalent variant into the yeast model *Saccharomyces cerevisiae* showed no direct impact on Gcn2 function, suggesting that the variant’s pathogenicity likely does not simply stem from altered GCN2 enzyme activity. Our yeast-based study helped narrow potential mechanisms, accelerating efforts to understand the variant’s role in colorectal cancer.

**Figure 1. Scoring for the effect of Gcn2 variants on Gcn2 activity:**
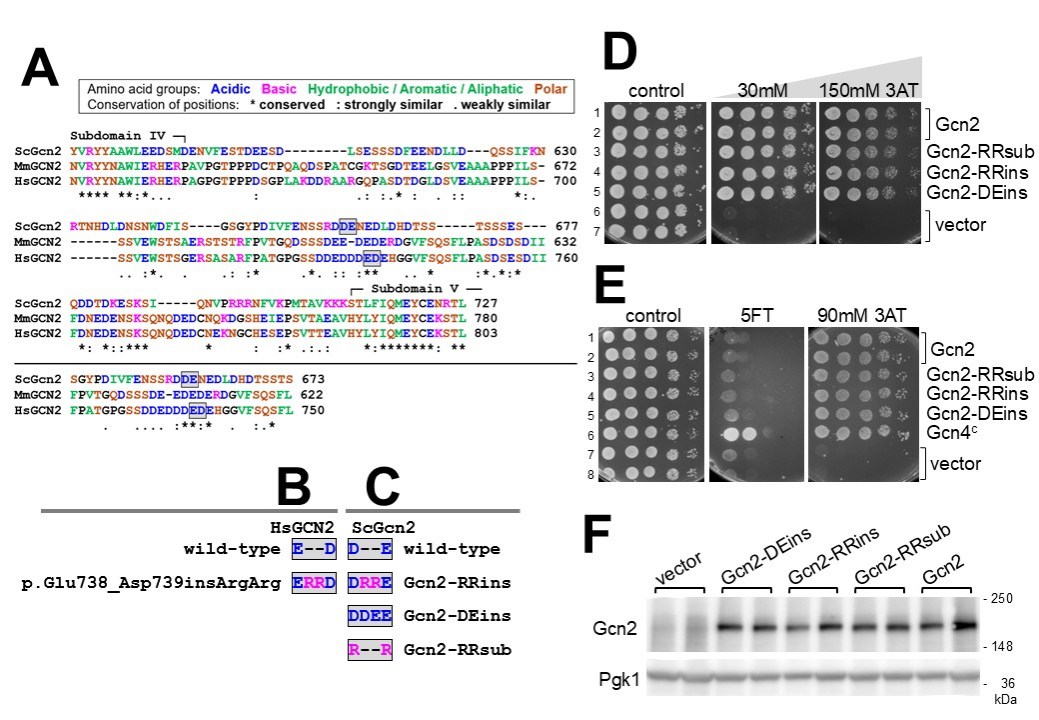
**(A)** A multi sequence alignment was performed with Gcn2 proteins from *Saccharomyces cerevisiae* (ScGcn2), mouse (MmGCN2) and human (HsGCN2) (accession numbers AAA34636.1, XP_017174398.1, KAI2573503.1), using Clustal Omega (Madeira et al. 2024), and a portion of the alignment - ranging from the protein kinase subdomain V to VI - is shown (top panel). The alignment was adjusted manually to illustrate that the level of similarity in the acidic region remains similar (bottom panel). Amino acids are colour coded as indicated by the key in the figure. **(B)** Amino acids 738 and 739 of wild-type human GCN2 are shown, as well as the amino acids for p.Glu738_Asp739insArgArg GCN2. **(C)** Amino acids 660 and 661 of wildtype yeast Gcn2 are shown, as well as the amino acid changes of the Gcn2 variants used in this study. **(D)** Yeast *gcn2Δ* strain H2557 harbouring the Gcn2 proteins as indicated, or lacking Gcn2 (vector), were subjected to semiquantitative growth assays using plates lacking 3-amino-2,4-triazole (3AT) (control), or containing 3AT at the concentration indicated. **(E)** Yeast *gcn2Δ* strain EMSY6053-3-1 harbouring plasmids as in (D), or a plasmid constitutively translating Gcn4 (Gcn4 ^c^ ) were subjected to the same assay as in (D), just that a plate was included containing 5-Fluorotryptophane. **(F)** Whole cell extracts of strains from (D) where subjected to western blotting to score for levels of Gcn2, and of Pgk1 as loading control.

## Description

GCN2 (General Control Nonderepressible 2) is a conserved serine/threonine-protein kinase that helps cells adapt to nutrient stress, particularly amino acid deprivation. Under starvation, GCN2 becomes activated and triggers a gene expression program that promotes survival by increasing the production of stress-responsive regulators like Gcn4 in yeast and AFT4 in mammals (Hinnebusch 2005).

GCN2, while largely dispensable in healthy cells, is critical for cancer cell growth and survival. Although mutations in the *EIF2AK4* gene (which encodes GCN2 in humans) have been found in some cancers, their functional significance is still unclear.

A variant in *EIF2AK4* was identified that features a double arginine (Arg) insertion in its protein kinase domain, specifically within the linker segment connecting subdomains IV and V ( Fig. 1A, boxed amino acids; Fig. 1B ). This GCN2 p.Glu738_Asp739insArgArg variant was significantly enriched in a cohort of early-onset colorectal cancer cases, where 33.3% of carriers developed colorectal cancer compared to 7% in the cancer-free control population (Zhang 2015). This suggests the double-Arg insertion may predispose to colorectal cancer, though direct evidence of causality remains to be established. To address this gap, we aimed to test whether the mutation affects GCN2 function using *Saccharomyces cerevisiae* , a model system highly amenable to genetic manipulation, allowing functional assays to be performed in a rapid and cost-effective manner (Pesic 2021). This was possible because GCN2 is highly conserved between yeast and humans (Castilho et al. 2014).

To determine the comparative location of p.Glu738_Asp739insArgArg in yeast Gcn2, multiple sequence alignments were performed between the Gcn2 proteins of *S. cerevisiae* , and human and mouse GCN2. The sequence of the linker segment is hardly conserved (Fig.1A, top alignment), however, the double-Arg insertion occurred within an acidic region present in mammalian GCN2 as well as yeast Gcn2. Notably, the acidic region showed low amino acid identity even between mouse and human GCN2, and alignment results varied depending on the algorithm and gap penalties used. An alternative, manually adjusted alignment yielded similar identity/similarity scores (Fig.1A, top vs. bottom), suggesting that overall charge, rather than precise sequence, is functionally important. Since Arg carries a positive charge, its insertion into the oppositely charged region may disrupt the functional integrity of this acidic region in Gcn2 activation. To test this in the yeast system, we inserted two Arg residues into the equivalent acidic region of yeast Gcn2 ( Fig. 1C ). In addition to the double Arg insertion, we designed a Gcn2 variant harbouring a double insertion of acidic amino acids to extend the acidic region, and another variant in which the amino acids neighbouring the above insertions were replaced by Arg (Fig.1C). The mutations were introduced by site-directed mutagenesis into an existing plasmid containing the *S. cerevisiae* *GCN2* gene under the control of its native promoter (Table I), and the resulting plasmids were introduced into yeast *gcn2Δ* strains, respectively.

To test whether these mutations affect Gcn2 activation, the corresponding yeast strains were subjected to semiquantitative growth assays (SQGA) using solid medium containing 3-amino-2,4-triazole (3AT), a compound that induces histidine starvation (Hilton, Kearney, and Ames 1965). As expected, cells lacking functional Gcn2 could not overcome starvation and therefore failed to grow, whereas cells harbouring functional Gcn2 grew under starvation conditions ( Fig. 1D, rows 1&2 vs 6&7). We found that, on 3AT medium, all the Gcn2 variants allowed cells to grow as well as the cells harbouring wild-type Gcn2, even in the presence of high 3AT concentrations ( Fig. 1D, rows 1&2 vs 3,4,5). This suggested that the mutations did not impair Gcn2 activation in response to starvation.

To test whether the mutations may render Gcn2 constitutively active, we repeated the SQGA but included plates containing 5-fluorotryptophan (5FT), a Trp analogue that does not elicit Gcn2 activation but is incorporated into proteins during translation (Miozzari, Niederberger, and Hütter 1977). The resulting production of nonfunctional proteins is deleterious to cell function, visible by lack of growth. As expected the strain containing wild-type Gcn2 was unable to grow in presence of 5FT ( Fig. 1E, rows 1&2). On the other hand, cells with a constitutively active Gcn2 - exhibiting increased expression of Trp biosynthetic genes and elevated Trp production - are less affected by 5FT, because the excess Trp outcompetes 5FT during the translation process. Here we resorted to cells harbouring constitutively translated Gcn4 (Mueller and Hinnebusch 1986), mimicking the downstream outcome of a constitutive active Gcn2, and as expected the strain was able to grow in presence of 5FT ( Fig. 1E, row 6). However, we found that none of the Gcn2 variants allowed yeast to grow on 5FT medium, as found for the strain expressing wild-type Gcn2 ( Fig. 1E, rows 1-5), supporting the idea that the Gcn2 mutations did not render Gcn2 constitutively active.

We did verify that the mutations in the Gcn2 variants did not affect Gcn2 protein levels, by performing immunoblotting assays, using antibodies against Gcn2 and against Pgk1 as loading control ( Fig. 1F ). Quantitative analyses revealed no statistically significant differences in protein levels between the Gcn2 variants and wild-type Gcn2.

In conclusion, our study suggests that modifications in the charged region of the linker between subdomains V and VI in yeast Gcn2 neither impaired nor enhanced the enzymatic activity. These modifications included the insertion of two basic Arg, the insertion of two acidic amino acids, and the replacement of the amino acids neighboring the insertion site with arginine (instead of adding additional residues). This seems to indicate that altering the charge in this acidic region does not affect Gcn2 activity. By analogy, the p.Glu738_Asp739insArgArg insertion in human GCN2 is unlikely to directly impact its enzymatic activity, suggesting that altered GCN2 activity is probably not the cause of colorectal cancer in this case. Possible scenarios are that the variant is correlative. Alternatively, if causative, it may require additional cellular alterations to exert a pathological effect, or act through human-specific mechanisms absent in yeast Gcn2 or yeast cells. While further work is needed to fully understand the role of the p.Glu738_Asp739insArgArg variant in colorectal cancer, our yeast-based findings help narrow down potential mechanisms and guide future investigations in mammalian systems.

## Methods

**Yeast strains and plasmids used**

Yeast strains and plasmids used in this study are summarized in Tables I and II . Plasmids expressing GCN2 variants were generated commercially via site-directed mutagenesis (Genscript, USA).

**Semi-quantitative growth assay (SQGA)**

Semi-quantitative growth assays were carried out as described previously (Ghuge et al. 2023). Briefly, 5 µL of 10-fold serially diluted saturated overnight cultures were transferred onto plates containing solid synthetic medium, and 3AT or 0.5 mM 5FT as indicated in the Figure. Plates were incubated at 30°C, and growth documented using a flatbed scanner.

**Generation of whole cell extracts and Western blotting**

Cell extracts were prepared from exponentially growing cells following published procedures (Lee et al. 2017), and whole cell extracts subjected to denaturing polyacrylamide gel electrophoresis (Lee et al. 2017; Anderson and Sattlegger 2021). Proteins were transferred onto a nitrocellulose membrane and the membrane cut horizontally at the 98 kDa band of the protein molecular weight marker (#LC5925, Thermo Fisher Scientific). Each part was probed with the respective primary antibody (Table III). Bound antibodies were visualised using horseradish peroxidase-conjugated secondary antibodies, Pierce ECL Western Blotting Substrate (#32209, Thermo Scientific, USA) and the ChemiDoc™ Imaging System (Bio-Rad, USA).

## Reagents

**Table I: Strains**

**Table:** 

**strain**	**genotype**	**source**
**Genetic background H1511**		
H1511	* MATα ura3-52 trp1-63 leu2-3,112, GAL2 ^+^*	(Foiani et al. 1991)
H2557	as H1511 but *gcn2Δ*	(Sattlegger and Hinnebusch 2000)
**Genetic background BY4741**		
MSY-WT2	*MATa leu2Δ0 met15Δ ura3Δ0*	(Sattlegger et al. 2004)
EMSY6053-3-1	as MSY-WT2 but *gcn2Δ::HisG*	(Sattlegger et al. 2004)

**Table II: Plasmids**

**Table:** 

**plasmid**	**protein**	**gene**	**marker**	**vector**	**source**
p722	Gcn2	*GCN2*	* Amp ^R^ , URA3 *	pRS316, ARS4/CEN6	(Wek et al. 1990)
pES809_p722-RRsub	Gcn2-RRsub	*gcn2* p.[D160R;E161R]	* Amp ^R^ , URA3 *	as p722	this work
pES811_p722-RRins	Gcn2-RRins	*gcn2* p.D160_E161insRR	* Amp ^R^ , URA3 *	as p722	this work
pES810ie809_p722-DEins	Gcn2-DEins	*gcn2* p.D160_E161insDE	* Amp ^R^ , URA3 *	as p722	this work
p238	Gcn4	* GCN4 ^a^*	* Amp ^R^ , URA3 *	YCp50, ARS1/CEN4	(Mueller and Hinnebusch 1986)

a The *GCN4* 3’ UTR lacks the uORF, leading to constitutive *GCN4* translation.

**Table III: Antibodies**

**Table:** 

**Antibody**		**Dilution**	**Order number, Source**
Anti- Gcn2	guinea pig polyclonal antibodies	1:1,000	(Visweswaraiah et al. 2011)
anti-Pgk1	mouse monoclonal antibodies	1:5,000	#459250, Thermo Scientific, USA
anti-guinea pig	horseradish peroxidase-conjugated goat antibodies	1:1,000	#2438, Santa Cruz Biotechnology, USA
anti-mouse	horseradish peroxidase-conjugated goat antibodies	1:10,000	#31430, Thermo Scientific, USA
